# Exploring the Career Motivations, Strengths, and Challenges of Autistic and Non-autistic University Students: Insights From a Participatory Study

**DOI:** 10.3389/fpsyg.2021.719827

**Published:** 2021-10-14

**Authors:** Chinnu Cheriyan, Sergey Shevchuk-Hill, Ariana Riccio, Jonathan Vincent, Steven K. Kapp, Eilidh Cage, Patrick Dwyer, Bella Kofner, Helen Attwood, Kristen Gillespie-Lynch

**Affiliations:** ^1^College of Staten Island, The City University of New York, Staten Island, NY, United States; ^2^The Graduate Center, The City University of New York, New York, NY, United States; ^3^School of Education, Language and Psychology, York St John University, York, United Kingdom; ^4^Department of Psychology, University of Portsmouth, Portsmouth, United Kingdom; ^5^Department of Psychology, University of Stirling, Stirling, United Kingdom; ^6^Center for Mind and Brain, University of California, Davis, Sacramento, CA, United States

**Keywords:** autistic, university, employment, participatory, discrimination, writing, strengths, stigma

## Abstract

Supports for the growing number of autistic university students often focus on helping them succeed in university. However, even educated autistic people experience discrimination and other challenges which can make it very difficult for them to obtain meaningful jobs. Little remains known about how universities can better support their autistic students and alumni in overcoming barriers to meaningful employment. In this participatory study, a team of autistic and non-autistic researchers asked autistic (*n* = 92) and non-autistic (*n* = 774) university students about their career aspirations, strengths they believe will help them succeed in their “dream jobs,” and obstacles they expect to encounter. Autistic participants’ top goal in attending college was to improve their career prospects. However, relatively few autistic students reported learning career-specific skills at university. Autistic students were more likely to seek an academic job and less likely to seek a career in healthcare than non-autistic students. Autistic students highlighted writing skills and detail orientation as strengths that could help them succeed in their dream jobs more often than non-autistic students. However, they were also more likely to expect discrimination, social, and psychological difficulties to stand in the way of their dream jobs. These findings suggest that universities should prioritize experiential learning opportunities to help autistic (and non-autistic) students develop employment-related skills while providing mental health supports. Universities should demonstrate their commitment to supporting diverse learners by seeking out and hiring autistic professionals and by teaching their own staff and employers how to appreciate and support autistic colleagues.

## Introduction

Higher education increases the likelihood that autistic people will obtain a job and be paid well for their work (Migliore et al., 2012; Ohl et al., 2017; Rast et al., 2020). Nevertheless, autistic people who successfully graduate from university remain less likely to be employed than university graduates with other disabilities (Association of Graduate Careers Advisory Services, 2019). A growing body of research examines strategies to help autistic students succeed in university (Kuder and Accardo, 2018). However, little remains known about how university staff can help autistic students and alumni obtain the jobs they deserve. A recent survey revealed that autistic people, their family members, employers, clinicians and researchers in the United States, Sweden, and Australia all agreed that a strong match between autistic peoples’ interests and job demands motivates autistic people to succeed in the workplace (Black et al., 2020). In order to help autistic students and alumni obtain jobs that are well matched to their interests, universities need to obtain more information about what autistic students’ employment interests are. To begin to address this gap, a participatory team of autistic and non-autistic researchers developed the current study to examine the career aspirations of autistic and non-autistic university students, strengths they believe will help them succeed in their “dream jobs,” and obstacles they expect to encounter.

### Why Do Autistic People Struggle to Get and Keep Jobs?

In stark contrast to the United Nations Convention on the Rights of Persons with Disabilities, which positions equal access to employment as a human right, autistic people around the world are chronically underemployed, even relative to people with other disabilities (Shattuck et al., 2012; Burgess and Cimera, 2014; Fasciglione, 2015; Farley et al., 2018; Scott et al., 2019). Meaningful employment can provide independence, social connections, and a sense of purpose and self-respect (Blustein, 2008; Hedley et al., 2017; Anderson et al., 2021). Barriers to employment, which are heightened among autistic people who come from lower-income families and/or are minorities (Eilenberg et al., 2019), have remained persistent over time (Burgess and Cimera, 2014). Among autistic people who do eventually obtain employment, many work at entry-level jobs that are not on par with their education and skills, receiving lower rates of pay than their non-autistic colleagues (Howlin et al., 2004; Roux et al., 2013; Baldwin et al., 2014; Coleman and Adams, 2018). In addition to pronounced difficulties *obtaining* employment, autistic people face challenges *maintaining* employment (Taylor et al., 2015; Chan et al., 2018). Although promising employment initiatives continue to emerge (e.g., Remington and Pellicano, 2019), they have primarily remained limited to specific employment sectors (e.g., IT and finance) and do not begin to address the full diversity of autistic people’s interests and skills (Lorenz and Heinitz, 2014; Bernick, 2021).

Despite often notable strengths (which may or may not include academic skills, attention to detail, high levels of empathy and moral clarity, creativity, focus, passion, honesty, loyalty, and logic), autistic people often face pronounced challenges obtaining and maintaining employment (Sperry and Mesibov, 2005; Lorenz and Heinitz, 2014; Dreaver et al., 2020; Stenning, 2020; Vincent and Fabri, 2020; Buckley et al., 2021; Fernandes et al., 2021). Social, executive functioning, sensory processing, and mental health differences; discrimination; insufficient autism understanding and needed supports; and poor work conditions contribute to the deeply problematic gap between the employment potential of autistic people and the reality of the employment outcomes they often obtain (Lorenz et al., 2016; Sarrett, 2017; Coleman and Adams, 2018; Farley et al., 2018; Black et al., 2020; Anderson et al., 2021; Bury et al., 2021; Buckley et al., 2021; Jones et al., 2021).

Milton (2012) proposed that non-autistic individuals struggle with taking the perspectives of autistic individuals and vice-versa. Thus, autistic individuals may struggle with adapting to “neurotypical” workplace social norms because the norms were not created with them in mind (Coleman and Adams, 2018). Indeed, when social difficulties arise for autistic employees, employers may often attribute these difficulties to characteristics of the autistic people themselves rather than seeking to improve the workplace (Bury et al., 2021). Autistic employees may also be more likely to attribute social challenges to their own internal characteristics rather than systemic issues. This tendency to attribute challenges to autistic individuals is at odds with increasing recognition that environmental factors play a key role in determining workplace outcomes (Harmuth et al., 2018; Black et al., 2019; Scott et al., 2019; Dreaver et al., 2020). Environmental barriers to succeeding at work, including insufficient support, unexpected changes, and sensory distractions have been associated with mental health issues, including burnout, among autistic educators (Wood and Happé, 2021).

Employment supports for autistic people often take a medical model orientation, focusing on ameliorating “deficits” within autistic individuals, rather than a neurodiversity-aligned approach which recognizes strengths associated with autism, views challenges as arising from dynamic interactions between people and their environments, and aims to systematically address environmental barriers (Singer, 2016; Scott et al., 2019). Yet workplace accessibility and autism understanding, social support, belief in one’s strengths, and the aforementioned strong match between autistic peoples’ strengths and interests and their job responsibilities have been identified by both autistic people and employers as crucial to employment success (Pfeiffer et al., 2017; Black et al., 2020; Dreaver et al., 2020; Wong et al., 2020; Pesonen et al., 2021).

However, little remains known about how strengths and interests influence the career choices of autistic university students. One of the few studies to examine the employment experiences of autistic university students used interviews with autistic students (*n* = 10) and alumni (*n* = 11) and focus groups with varied stakeholders (*n* = 58), finding that autistic students and alumni often faced extreme challenges obtaining stable jobs (Vincent and Fabri, 2020). Participants described inaccessible hiring practices (e.g., opaque job advertisements, anxiety-provoking interviews wherein autistic students struggled to adapt to “neurotypical” norms, and concerns that disclosing a diagnosis would lead to discrimination). Although university career services were often described as helpful, others described career services as underfunded and under-informed about autism. Pesonen et al. (2020) also documented a desire for more accessible, individualized, and hands-on university career support among 30 autistic university students in four European countries.

### Research Aims and Hypotheses

To help universities better support autistic students in achieving their career goals, we compared autistic and non-autistic students’ hopes and concerns about employment. We expected autistic students to more often cite a desire to help others and passion for their interests as reasons for seeking a career than non-autistic students. The first and second author developed these hypotheses based on prior research and their own experiences engaging with autistic mentees and mentors within a participatory mentorship program they were mentors within. Prior research suggests that autistic people may feel intensely for others, particularly those who are vulnerable, and may help others more than non-autistic people do (Smith, 2009; Paulus and Rosal-Grifoll, 2017; Stenning, 2020). Passion for one’s interests is also commonly reported as an autism-related strength (e.g., Lorenz and Heinitz, 2014).

We expected autistic students to more often describe detail orientation and writing skills as assets that could help them get their dream jobs than non-autistic students. Many autistic people are able to recognize details that may remain hidden to “neurotypical” individuals (e.g., Dakin and Frith, 2005; Lorenz and Heinitz, 2014). Autistic university students also exhibited enhanced writing skills relative to their non-autistic peers at one university (Gillespie-Lynch et al., 2020). Similar patterns have been observed in more generalizable samples: incoming autistic university students in the Netherlands outperformed their peers on the Dutch Language Proficiency Test (Bakker et al., 2019), as did autistic students in the United States on the verbal SAT (Fernandes et al., 2021).

We expected autistic students to describe discrimination as a hurdle more often than non-autistic students given that autistic people typically identify stigma as the most consequential barrier to employment (Black et al., 2020). We also expected autistic students to describe focus and social skills as potential challenges more often than non-autistic students (Lorenz and Heinitz, 2014; Scott et al., 2019).

## Methods

### Community Involvement

The research described in this report was conducted by a participatory group of autistic and non-autistic researchers, including the authors of this paper and a larger group that collaborated more distally. Collaborators included leaders within a participatory mentorship program for autistic and non-autistic university students, Project REACH, and members of the College Autism Network (CAN), an online community of individuals dedicated to advocacy and research related to autistic university students. Contributors to this paper include four autistic co-authors (one academic, one doctoral student, one then-undergraduate and current graduate student, and one undergraduate) and six non-autistic group members (two undergraduates with other diagnoses; one then-doctoral student, who led study development as part of her dissertation research; and three academics).

Autistic and non-autistic co-authors collaboratively developed and revised study measures and hypotheses by co-writing a shared google document. Research questions and hypotheses were collaboratively developed at the top of the google doc, to promote transparency about research goals and member contributions. Transparency is central to the guidelines that AASPIRE, the first participatory autism research group (Jivraj et al., 2014), provided to help researchers practice sound participatory autism research (Nicolaidis et al., 2019).

Survey questions were developed based on collaborators’ experiences and knowledge of the literature. Questions were iteratively revised until collaborators were satisfied with their scope and clarity. We began developing measures in April of 2018 and continued revising until recruitment began in February of 2019. Most revisions occurred asynchronously, via edits and/or comments in the study google doc. However, a core group of collaborators, leaders in the aforementioned mentorship program, discussed and polished research questions, hypotheses, and measures during synchronous meetings which occurred in-person or virtually (depending on location and/or preference) via Skype using speech or text chat as preferred. These meetings were held approximately once every 6 weeks. Guided by AASPIRE’s guidelines (Nicolaidis et al., 2019), we strove to use flexible communication modalities, to provide sufficient processing time, to develop strategies for power sharing, and to disseminate findings collaboratively. The survey itself was posted on the Open Science Framework before recruitment began. However, the hypotheses described in this report were developed by the first two authors before they gained access to the data.

### Survey Development and Recruitment

Data were collected via online surveys hosted on Qualtrics from February to October 2019. Autistic and non-autistic participants were generally recruited via different mechanisms. Autistic participants were primarily recruited via snowball sampling using collaborators’ networks (e.g., CAN, social media, and university contacts). Given that convenience samples are always biased toward people who are informed about and decide to participate in a study, we share the exact recruitment invitation here, “Are you a current university student? Are you autistic? Help us learn more about autism and your experiences with higher education!” Interested autistic participants contacted the third author via an institutional email address (to confirm student status) and received a link to participate. They received a $20 Amazon gift card.

Members of the primarily non-autistic comparison sample were invited to participate in a study entitled “community conceptions of diversity” through psychology subject pools at two universities in the United States: one in the Midwest with highly selective admissions criteria and one in the Northeast which is not selective. They received no other information about the study, besides its title, when deciding if they wished to participate. Eight participants recruited via this mechanism identified as autistic. Participants recruited from subject pools completed the questions that are the focus of this report at the beginning of a survey before participating in an autism training. They received academic credit for participating.

### Participants

All participants completed an IRB-approved consent form prior to beginning the study (see Table 1 for participant characteristics). Autistic university students (*n* = 92; 53.3% male) representing eight countries (*n* = 68 from the United States, *n* = 15 from the United Kingdom) and about 50 institutions and non-autistic students from two institutions (*n* = 774; 35.0% male) participated in this study. Although most of the autistic participants (*n* = 84) were recruited to our CAN survey, eight autistic participants were recruited to the conceptions of diversity survey via the aforementioned subject pools.

**TABLE 1:** Demographics.

	**Autistic *n* = 92(%)**	**Non-autistic *n* = 774(%)**
**Age**		
18–24	75.0	92.2
25–34	17.4	2.8
35–44	4.3	0.3
45+	3.3	0.5
**Gender**		
Male	53.3	35.0
Female	34.8	61.9
Non-binary	11.9	0.6
**Race/Ethnicity**		
White	77.2	41.5
Black/African	6.5	19.6
Asian	10.9	18.3
Latinx	7.6	25.5
Middle Eastern	–	5.9
Indigenous/Pacific Islander	6.5	2.5
Other	12	0.8
**Area of study**		
STEM	48.9	53.4
Social Science	21.7	19.8
Medical	3.3	11.4
Humanities	27.2	4.7
Education	5.4	6.2
Business	3.3	8.3
Undecided/Liberal Arts	7.6	4.9

Students recruited through the CAN survey provided information about their diagnosis and completed the Ritvo Autism and Asperger Diagnostic Scale (RAADS-14) (Eriksson et al., 2013), a self-report screener for autism in adulthood (α = 0.84). We included this measure to determine what proportion of our autistic sample would be classified as likely to be autistic using a commonly used autism screener. This brief measure provides both a dimensional and a categorical rating of autism likelihood, has relatively strong psychometric properties, and includes a focus on sensory differences, which is often lacking in autistic trait measures (Baghdadli et al., 2017).

Although some members of the autism community view autism screening and trait measures as overly deficit-oriented, only 3 of the participants in the current study critiqued the RAADS-14 when asked if they like to give feedback throughout the survey. Two participants indicated that the response scale was not precise enough. For example, one participant wrote, “For the questions on Autistic behaviors that consisted of a statement to which I had to specify whether it was true, true only now, true when I was younger, or never true, I found these somewhat difficult to answer. They are essentially a binary true/false question with a temporal component, so I cannot answer to what extent I agree with the statement. Some statements were very true when I was younger but are only mildly true currently.” One participant specifically critiqued the focus on challenges, “Please phrase questions more autism-positive.”

For the 84 students who were recruited through the CAN survey, autism identification was confirmed using self-report of an autism diagnosis by a clinician (*n* = 81) or self-report of autism identification without a formal diagnosis (*n* = 3). Participants reported mean RAADS-14 scores of 27.7 (*SD* = 9.15). Seventy-eight participants reported RAADS-14 scores at or above the suggested cut-off for probable autism of 14; the six who did not meet the cut-off all reported having a formal autism diagnosis.

Students recruited through the subject pool indicated the relationships they had experienced to autism (including being autistic themselves). A total of 782 subject pool students (*n* = 624 from the school in the Northeast; *n* = 8 self-identified as autistic) provided demographic information and completed the open-ended questions about employment goals that are the focus of this report.

Autistic participants were more likely to identify as only white (62.0%), male (53.3%), graduate students (20.7%), and humanities majors (27.2%) than non-autistic students (31.9% only white; 35.0% male, 5.7% graduate students, and 4.7% humanities majors; *p*s < 0.001; see Table 1).

### Survey Questions

In addition to demographic questions, autistic and non-autistic participants were asked the following open-ended questions (developed through the collaborative process described above):

(1) What is your course of study/major? If you are undecided, please let us know what majors/fields of study you are considering.
(2) What type of job do you hope to get after you graduate?
(3) Why is this job of interest to you?
(4) What skills do you have that could help you succeed in your dream job?
(5) What challenges might you face getting or keeping your dream job?

Only autistic participants recruited through the snowball sampling were asked the following open-ended and closed-ended questions:

(1) What goals do you hope university will help you achieve?
(2) Has your experience in university helped you develop work-related skills? (options: yes or no)
(3) What work-related skills have you developed so far in university?
(4) What is the job you held for the longest time?
(5) Did you receive specialized supports due to autism at your job (options yes/no)?
(6) When do you tell potential or current employers about your autism? (option to select any combination of: on your resume/CV, on your job application, during the interview, soon after being hired, if an issue arises, when I get close to people at work, I only partially disclose, I don’t tell people at work anything about autism, other).

Participants were given opportunities to provide feedback on survey questions, “Is there anything you would like to say about the questions so far? Let us know if some questions were unclear or if there were things we should have asked about but didn’t. Your feedback will help us improve.” Students generally provided positive feedback about the employment-related questions, e.g., “Everything has been clear so far, I have had no problems.”

### Qualitative Coding and Data Analysis

Responses to open-ended questions were coded using content analysis by pairs of coders who developed codes based on patterns in the data (and hypotheses) and obtained reliability of 80% or higher on all codes. We elected to use content analysis because we wished to compare the frequency at which different strengths, barriers, and motivations emerged from responses. Content analysis, a method of coding raw messages (e.g., text or images) into a classification scheme, first emerged in the 18th century and is increasingly used (Kondracki et al., 2002; Hsieh and Shannon, 2005). It is a broad approach to deriving meaning about a phenomenon that varies along two primary spectrums: manifest (or apparent on the surface) to latent (deeper implied meanings) themes and inductive (data driven) to deductive (theory driven). The coding used in this study focused on manifest meanings. Most codes were developed inductively through an independent review of the data by the first two co-authors. However, codes used to address hypotheses and/or to align coding with existing classification systems were primarily deductive.

The two first authors coded majors into: STEM, humanities, education, business, liberal arts/undecided, and other. They coded dream jobs using O^∗^NET’s categories (National Center for O^∗^NET Development, 2021), adding codes for helping professions^1^, faculty/academia, and entrepreneurs. For responses about why the job is of interest, they coded motivation as intrinsic (with subcodes helping others, passion, and knowledge) or extrinsic (with subcodes financial security and fame, the latter only occurred once in each sample so is not considered further). For skills that could help students succeed in their dream job, they coded knowledge, motivation, intelligence, detail orientation, executive functioning (with subcodes focus and reliability), patience, social communication (with subcodes social skills, empathy, and writing), and work ethic. For challenges obtaining or maintaining one’s dream job, they coded discrimination, motivation, psychological difficulties, competition, executive functioning (with subcodes focus and organization), social communication (with subcodes social skills, empathy, and writing), financial problems, academic issues, and work ethic (see Appendix A for full coding schemes).

A different pair of student co-authors (both autistic) developed coding schemes for the first two open-ended questions asked of only autistic students and obtained reliability. They coded the goals participants hoped college will help them achieve into the following major categories: academic progression, career prospects, personal development, interpersonal, community-oriented reasons, and financial reasons. They coded the skills participants felt they had developed so far at university into these major categories: career skills, academic skills, personal development, and interpersonal (see Appendix A).

We conducted chi-square tests of independence to compare autistic and non-autistic participants’ career goals, motivations, and anticipated career-related strengths and challenges. Following Benjamin and Berger’s (2019) recommendation, we use an alpha level of 0.005 and consider *p* values between 0.005 and 0.05 suggestive. To examine if significant group differences were attributable to other differences between the samples besides autism, we conducted follow-up binary logistic regressions with the following characteristics that differed across samples included as predictors: being male, white, a graduate student, and/or majoring in humanities^2^.

## Results

### What Employment Skills Have Autistic Participants Learned at University?

Most autistic participants recruited through the snowball sampling (62%) had already been employed, mostly in entry-level jobs. Autistic participants’ top goals in attending university were to improve their career prospects, followed by academic progression, interpersonal and personal development (see Appendix B for code frequencies and illustrative quotes). When asked if they had learned employment-related skills at college, most autistic participants (76%) said they had. When asked what employment-related skills they had learned so far at university, autistic students most often highlighted personal development, followed by academic skills, interpersonal skills and lastly career-specific skills (see Appendix C for code frequencies and illustrative quotes). Career-specific skills were defined as participants specifically indicating employment-related activities and experiences in their responses.

Students expressed diverse perspectives about disclosing their autism at work: 2% indicated that they did or would disclose on their CV, 5% during the interview, 2% soon after being hired, 4% if an issue arises, 5% when they get close to people at work, 4% only partially disclose, 20% indicated that they don’t tell anyone at work anything about autism, and 13% selected other, typically adding that the decision is context-dependent. The rest of the participants (45%) selected multiple options, often indicating that the decision is highly context-dependent. For example, one student wrote, “For my current job, I disclosed much earlier because my autistic identity is integral to my work as a student program coordinator and researcher.” However, working in an autism field does not always lead to disclosure. Another student wrote, “Because I worked with autistic individuals I did not feel comfortable disclosing because I didn’t want to be treated differently.”

### What Jobs Do Students Want?

Autistic students were more likely to seek an academic job than non-autistic students (*p* < 0.001; see Table 2). They were less likely to seek a helping career and specifically a career in healthcare (*p*s < 0.001). A binary logistic regression showed that pursuing academia was predicted by being autistic and a graduate student (suggestive; see Table 3). Males and humanities majors (suggestive) were less interested in helping careers, although being autistic also contributed suggestively (Table 3). Interest in a career in healthcare in particular was predicted by being non-autistic, female and not a graduate student (Table 3).^3^

**TABLE 2:** Career goals of autistic and non-autistic students.

**Code**	**Autistic**	**Non-autistic**	***p* value**
**Helping Profession**	**39%**	**60.7%**	**<0.001**
**Academic Job**	**11%**	**0.3%**	**<0.001**
Entrepreneur	0%	3.2%	0.10
Management	2%	1%	1.00
*Business/Finance*	*0%*	*4%*	*0.026*
Computer/Math	11%	1%	0.026
Architecture/Engineering	2%	4%	0.57
*Science*	*17%*	*1%*	*0.015*
Social Service	2%	1%	0.42
*Education/Library*	*17%*	*3%*	*0.02*
*Arts/Media/Sports*	*10%*	*4%*	*0.006*
**Healthcare**	**9%**	**41%**	**<0.001**
Protective	0%	3%	0.15
Don’t know	10%	5%	0.10

Motivations underlying career goals of autistic and non-autistic students.			

**Code**	**Autistic**	**Non-autistic**	***p* value**

Intrinsic Interests	85%	83%	0.77
*SC: Help Others*	*28%*	*41%*	*0.02*
SC: Passion	38%	33%	0.42
*SC: Knowledge*	*10%*	*4%*	*0.03*
Extrinsic	9%	11%	0.60
SC: Financial Security	4%	8%	0.30

*Italics highlight evidence suggestive of a group difference. Bolded indicates group difference.*

**TABLE 3:** Binary logistic regression predicting seeking an academic career.

**Nagelkerke’s *R*^2^ (0.39)**	**OR [95% CI]**
Autistic	53.08 [9.91, 284.40]*
Male	0.89 [0.24, 3.23]
White	0.26 [0.06, 1.02]
Graduate student	6.26 [1.65, 23.73]^
Humanities major	0.65 [0.11, 3.68]

Binary logistic regression predicting seeking a helping career.	

**Nagelkerke’s *R*^2^ (0.18)**	**OR [95% CI]**

Autistic	0.54 [0.32, 0.90]^
Male	0.23 [0.17, 0.31]*
White	0.95 [0.69, 1.30]
Graduate student	0.76 [0.43, 1.33]
Humanities major	0.45 [0.25, 0.81]^

Binary logistic regression predicting seeking a career in healthcare.	

**Nagelkerke’s *R*^2^ (0.15)**	**OR [95% CI]**

Autistic	0.15 [0.06, 0.36]*
Male	0.40 [0.29, 0.56]*
White	0.79 [0.58, 1.09]
Graduate student	0.36 [0.17, 0.74]*
Humanities major	0.50 [0.25, 1.03]

*p < = 0.005.

*^0.005 < *p* < 0.05.*

### Why Are Students Seeking Specific Jobs?

Autistic and non-autistic university students expressed similar reasons for pursuing their dream jobs. Contrary to our hypothesis that autistic students would be more motivated by their interests to pursue their dream jobs, the vast majority of both autistic and non-autistic students were driven by intrinsic interests (Table 2). Contrary to our hypothesis that autistic students would more often cite a desire to help others as a reason for seeking a career than non-autistic students, evidence suggested that autistic students were slightly *less* likely to highlight a desire to help others as a career motivation (*p* = 0.02). Although this finding did not meet our criteria for significance, we conducted a follow-up binary logistic regression as it was in the opposite direction of our expectation. This difference was attributable to males being less interested in helping others (OR = 0.33, 95% CI [0.24, 0.45]; *p* < 0.001) rather than autism, race, graduate status, or major (*p*s > 0.19).

### What Skills Do Students Think Will Help Them Succeed in Their Dream Jobs?

Autistic students were more likely to highlight writing skills (*p* < 0.001) and detail orientation (*p* = 0.003) as skills that would help them get their dream job than non-autistic students (Table 4; see Appendix D for illustrative quotes). A binary logistic regression with the aforementioned predictors (*p*s > 0.13) showed that only being autistic was associated with heightened detail orientation (OR = 6.62, 95% CI [2.17, 20.19], *p* = 0.001; Nagelkerke’s *R*^2^ = 0.08). Similarly, writing skills were predicted by being autistic (OR = 23.55, 95% CI [7.27, 76.33], *p* = 0.001) or a humanities major (suggestive; OR = 3.19, 95% CI [1.10, 9.30], *p* = 0.033; Nagelkerke’s *R*^2^ = 0.31) but no other predictors (*p*s > 0.46).

**TABLE 4:** Challenges and strengths autistic and non-autistic students expect in securing their career.

	**Challenges**			**Strengths**		
**Code**	**Autistic**	**Non-autistic**	***p* value**	**Autistic**	**Non-autistic**	***p* value**
**Discrimination**	**15%**	**1%**	**<0.001**	–	–	–
**Academic Issues**	**7%**	**34%**	**<0.001**			
*Psychological Difficulties*	*16%*	*7%*	*0.008*	*–*	*–*	*–*
Competition	13%	17%	0.38			
Financial Problems	7%	7%	1.00			
Motivation	3%	7%	0.26	14%	16%	0.76
Work Ethic	1%	1%	0.72	*4%*	*11%*	*0.046*
**Social Communication**	**28%**	**7%**	**<0.001**	33%	42%	0.09
**SC: Social Skills**	**25%**	**6%**	**<0.001**	*16%*	*26%*	*0.06*
SC: Empathy	1%	0.1%	0.21	7%	13%	0.07
**SC: Writing**	0%	0.1%	1.00	**16%**	**1%**	**<0.001**
*Executive Functioning*	*9%*	*3%*	*0.015*	12%	9%	0.46
SC: Focus	1%	1%	0.43	3%	2%	0.48
*SC: Organization*	*7%*	*2%*	*0.02*			
SC: Reliability				7%	7%	0.41
*Knowledge*	*–*	*–*	*–*	*28%*	*28%*	*0.45*
Intelligence	–	–	–	19%	13%	0.20
*Detail Orientation*				*8%*	*2%*	*0.003*
*Patience*				*2%*	*11%*	*0.003*
Don’t know	7%	5%	0.63	3%	2%	0.24

*Italics highlight evidence suggestive of a group difference.*

*Bolded indicates group difference.*

Non-autistic students were more likely to describe patience (*p* = 0.003) as a job strength than autistic students. A binary logistic regression showed that this difference was attributable to males (OR = 0.28, 95% CI [0.15, 0.51], *p* < 0.001; Nagelkerke’s *R*^2^ = 0.08) being less likely to report patience rather than autism (*p* = 0.052). No group differences were observed in the degree to which motivation, executive functioning, or intelligence were noted as career-skills (*ps* > 0.20).

### What Challenges Do Students Expect to Encounter Seeking and Keeping Dream Jobs?

When asked what challenges they expect to face obtaining or maintaining their dream job, autistic students were more likely to highlight discrimination (*p* < 0.001) and social challenges (*p* < 0.001) with evidence suggestive of associations between autism and psychological difficulties (*p* = 0.008) and executive functioning (*p* = 0.015; see Table 4 and Appendix E for illustrative quotes). Follow-up binary logistic regressions revealed that expecting discrimination (OR = 24.57, 95% CI [8.78, 66.78], *p* < 0.001; Nagelkerke’s *R*^2^ = 0.26) and social difficulties (OR = 5.90, 95% CI [3.19, 10.91], *p* < 0.001; Nagelkerke’s *R*^2^ = 0.10) were only predicted by being autistic. Psychological difficulties were predicted by being autistic (OR = 2.98, 95% CI [1.47, 6.06], *p* < 0.001) and not a male (OR = 0.35, 95% CI [0.19, 0.64], *p* < 0.001; Nagelkerke’s *R*^2^ = 0.06).

Non-autistic students were more likely to report academic challenges as barriers to their dream job than autistic students (*p* < 0.001). Academic difficulties were predicted by being not autistic (OR = 0.12, 95% CI [0.05, 0.32], *p* < 0.001) or a humanities major (suggestive; OR = 0.38, 95% CI [0.17, 0.88], *p* = 0.023; Nagelkerke’s *R*^2^ = 0.08). Autistic students were slightly more likely to note executive functioning, but not specifically focus, as a challenge (*p* = 0.015; suggestive). No differences in motivation, competition, work ethic, or financial barriers were noted (*p*s > 0.25).^4^

## Discussion

Autistic students’ primary goal in entering university was to advance in their careers. Although most reported that university helped them develop employment-related skills, most of the career-related skills they described developing were general skills, like self-understanding and academic development, rather than hands-on opportunities to practice applying for and/or succeeding in the workplace. Skills that specifically targeted a career were *least* likely to be highlighted among the work-related skills autistic students felt they developed at university. To align curriculum with autistic students’ goals in seeking higher education, universities should provide more targeted support for career-related skill development for autistic students, such as internships and other forms of experiential learning. Such support should be open to students who are and are not autistic, given that many autistic students are not comfortable disclosing that they are autistic and autism is underdiagnosed among people who are not white males (Happé and Frith, 2020).

Difficulties applying one’s education to obtain a job one is happy with are far from specific to autistic students. Concerns about the degree to which universities are preparing students more generally to succeed in the workforce have led to calls for more sandwich courses and internships (and greater integration of internships with academic requirements), focused technical education delivered by industry professionals (e.g., data analysis), and more opportunities for students to try out professional tools and roles in the classroom (e.g., UC Berkeley’s innovative student led courses, DeCal; Cleary and Van Noy, 2014; Brooks and Youngson, 2016; Frazee and Level, 2018). Although a misalignment between students’ educational preparation and the demands of the workforce is a broader concern, autistic people face much more pronounced barriers obtaining meaningful work than other students. Indeed, autistic participants were much more likely to expect discrimination to stand in the way of their dream jobs than other students. Unfortunately, this expectation is not inaccurate; discrimination has emerged as a consistent barrier to autistic employment across varied studies (e.g., Scott et al., 2019; Black et al., 2020). A key factor that is known to help other marginalized groups overcome misconceptions about their ability to succeed in a field (e.g., women in STEM) is access to educators and other role models like them in the fields they hope to enter (Cheryan and Plaut, 2010). Therefore, one important way that universities can encourage autistic students to keep striving toward their dream jobs is to hire autistic educators and staff. However, universities rarely prioritize attracting and supporting neurologically diverse staff (Brown and Leigh, 2018; Jones, 2021). Insufficient efforts to recruit and support autistic university staff is particularly problematic given that many autistic people may be drawn to academic careers, as was evident in our study. A central recommendation derived from this work is that universities should act as examples of inclusive hiring practices for the broader community, rather than recapitulating existing inequalities.

Autistic (and other) students need opportunities to connect with diverse industries through their universities. Students more generally often call for more contact with industry professionals, including alumni who can share their own experiences in the workforce (Donald et al., 2018). Attempts to create job opportunities for autistic students must be much broader than their current focus primarily on the tech sector. Participants in our study expressed a wide variety of career interests. Unexpectedly, autistic students were less likely to seek careers in helping fields, particularly healthcare, than non-autistic students. Only 11% sought careers in IT fields, clearly supporting the need for greater diversification of autism employment initiatives.

Contrary to our hypothesis (and inconsistent with the stereotype that autism is defined by “fixated interests”), both autistic *and* non-autistic students were similarly highly guided toward their dream jobs by intrinsic interests. Evidence also unexpectedly suggested that autistic students were slightly *less* likely to be drawn to their dream jobs by a desire to help others than non-autistic students. A follow-up analysis revealed that this difference was attributable to men being less motivated to help others through their work than women. This gender difference has been documented previously (e.g., Morgan et al., 2001).

Extending findings from larger-scale studies focused on the university experiences of autistic students to the employment domain (Bakker et al., 2019; Sturm and Kasari, 2019; Fernandes et al., 2021), autistic students were more likely to highlight writing skills as a career-related strength and less likely to highlight academic difficulties as a challenge than non-autistic students. Aligning with findings from the broader autism employment literature, which has not typically distinguished between university students and others analytically (Scott et al., 2019; Black et al., 2020), autistic students were more likely to report detail orientation as a career-related strength but expected to encounter challenges obtaining and maintaining their dream jobs due to discrimination, social difficulties, and psychological difficulties. Likely due to concerns about discrimination, few autistic participants reported proactively disclosing their autism.

### Limitations and Future Directions

Our non-autistic comparison group was not well-matched to our autistic group and autistic participants were predominantly white males. Findings require replication with larger and more generalizable samples. An autistic graduate degree holder responded to our preprint by writing, “this work is absolutely needed… (but) it hit me hard to find there was no representation in the sample for someone like myself.” They highlighted the importance of improving representation of autistic people from different cultural backgrounds given that discrimination and access to diagnoses vary across cultures. Given the pronounced limitations in generalizability imposed by our unrepresentative convenience samples, we follow their advice by including a link to a feedback form so readers can share their insights about how to improve future work in this area: https://bit.ly/EmploymentPaperFeedback.

Like most prior work focused on autistic university students (e.g., the large body of work focused on the National Longitudinal Transition Study or the Freshman Survey), we examined students’ self-perceptions rather than objective indicators of strengths and challenges. Work is needed that examines whether self-reports align with objective indicators of academic and employment success. Such work should examine if perceived strengths and challenges shape career goals and trajectories longitudinally.

## Conclusion

These findings indicate that programs aiming to help autistic university students obtain meaningful jobs should provide strengths-focused supports to help all students, and particularly those who face pronounced obstacles gaining meaningful employment, develop employment-related skills at university, while also providing mental health supports. Universities can begin to address the barriers their autistic students expect to face obtaining their dream jobs by proactively seeking out and hiring autistic professionals, by providing hands-on learning experiences co-designed with potential employers to help students develop the skills employers are seeking, and by teaching their own staff and community partners how to appreciate and support autistic colleagues. Employer education programs should focus on making workplaces more inclusive by combatting discrimination and by changing the hiring process and work environment to better suit the needs of autistic individuals. Although the systemic barriers that make it hard for autistic people to find jobs that allow them to express their strengths can feel like a permanent part of our society, they are shaped by cultural and economic forces that are changing (Grinker, 2020) and which we can help to change.

## Data Availability Statement

The original contributions presented in the study are included in the article/Supplementary Material, further inquiries can be directed to the corresponding author/s.

## Ethics Statement

The studies involving human participants were reviewed and approved by The College of Staten Island Institutional Review Board. The patients/participants provided online informed consent to participate in this study. Written informed consent was obtained from the individual(s) for the publication of any potentially identifiable images or data included in this article.

## Author Contributions

CC and SS-H developed the hypotheses and coding schemes and coded the majority of the data (i.e., questions asked of both autistic and non-autistic students). CC wrote numerous initial drafts of this manuscript for her honors thesis. AR led survey development and recruitment in partnership with a larger participatory research group as part of a larger study about autistic university students as part of her doctoral dissertation. CC, JV, SKK, EC, PD, and BK assisted with survey development, recruitment, and/or offered feedback on coding schemes. SKK, JV, BK, PD, AR, and EC edited this manuscript. BK and HA analyzed responses to develop a coding scheme about the goals autistic participants hope college will help them achieve and developed so far at university. As CC’s and AR’s advisor, KG-L guided survey, coding scheme and thesis development, conducted the final analyses and literature review, wrote the final draft of this manuscript, and addressed reviewer feedback. All authors contributed to the article and approved the submitted version.

## Conflict of Interest

The authors declare that the research was conducted in the absence of any commercial or financial relationships that could be construed as a potential conflict of interest.

## Publisher’s Note

All claims expressed in this article are solely those of the authors and do not necessarily represent those of their affiliated organizations, or those of the publisher, the editors and the reviewers. Any product that may be evaluated in this article, or claim that may be made by its manufacturer, is not guaranteed or endorsed by the publisher.

## Footnotes

1 The first two authors defined helping professions based on their own understanding of how central helping others is to different jobs. Obviously, this classification is a bit of an overgeneralization, as any career can be focused on helping others depending on the priorities of the person in said career.

2 We did not include age in these analyses because the question about age was phrased differently in the two samples. However, including a binary age variable (emerging adult or not) in all binary logistic regressions, did not change the pattern of findings.

3 These predictors of seeking a career in healthcare remained significant when the stated desire to help others through one’s work (OR = 4.08; *p* < 0.001) was included as a predictor.

4 All group differences, except those pertaining to detail orientation, patience, and psychological difficulties, remained apparent when comparisons focused only on non-autistic students from the more selective school.
