# Supplementary material for: Exploring the Career Motivations, Strengths, and Challenges of Autistic and Non-autistic University Students: Insights From a Participatory Study
Source: Front Psychol. 2021 Oct 14;12:719827. doi: 10.3389/fpsyg.2021.719827 (PMC8568013; doi:10.3389/fpsyg.2021.719827)
Supplement: Supplementary file 1 [file Table_1.docx]

**Appendix A**

**Full Coding Schemes**

**Question to code: What type of job do you hope to get after you graduate?**

Source: ONET [Main Page](https://www.onetcenter.org/database.html#individual-files), [Manual](https://www.bls.gov/soc/2018/soc_2018_manual.pdf), [Search O-NET](https://www.onetonline.org/find/)

- First two numbers based on ONET

We will look up each profession using ONET’s search function and input the initial two numbers of the code here. However, we will code “Healthcare Support Occupations” (31) together with “Healthcare Practitioners and Technical Occupations” as 29. Individuals who put down two occupations will receive both codes. Individuals whose dream jobs fit two separate codes (i.e.: academia, 19 and 25) would receive only one of the codes if they indicate being interested in that specific aspect of that profession or both codes if they do not indicate a preference or indicate an interest in both.

- Helping professions

“Occupations that provide health and education services to individuals and groups, including occupations in the fields of psychology, psychiatry, counseling, medicine, nursing, social work, physical and occupational therapy, teaching, and education” ([APA](https://dictionary.apa.org/helping-professions)) will be included here. We will also include researchers if they specify wanting to help others as one of their reasons for choosing the job.

- i.e.: participants coded as 21, 25, or 29 will automatically receive this code; participants coded as 19 will receive this code if they are interested in working as a psychologist or have indicated that they want to help others as a motivation for choosing to work as a researcher.
- College/University Faculty or Academia

Participants that indicate that they would like to become members of academia will receive this code.

- Entrepreneurs

Participants that would like to own their own business would receive this code regardless of whether they fall in the management occupations category according to ONET.

- IDK
- N/A
- Other

**Question to code: Why is this job of interest to you?**

- Intrinsic

Examples: general interest in the subject; expressing a job-related desire, such as working with a specific population, desiring independence, or wanting a specific work style.

- (SC) Helping others

The participant expresses a desire to make a difference or help others.

- (SC) Intrinsic Passion

The participant expresses being enthusiastic or passionate about their career of choice or uses words such as “like,” “love,” or “enjoy” in reference to it.

- (SC) Knowledge

The participant mentions experience gained from working a job, having an internship in the field, or from knowledge gained in college. Also applies if the participant states that they have a specific skill related to their dream job. Any experience, knowledge, or skills mentioned have to directly relate to their job of interest. The participant provides their job of interest in answer to “What type of job do you hope to get after you graduate?”

- Extrinsic

Examples: avoiding punishment, or other non-intrinsic factors.

- (SC) Financial Security

The participant states wanting wealth, job security, or other financial benefits as a reason for selecting their profession.

- (SC) Fame

The participant cites the desire for fame or attention as a reason for selecting their career.

**Question to code: What skills do you have that could help you succeed in your dream job?**

- Knowledge

The participant mentions experience gained from working a job, having an internship in the field, or from knowledge gained in college. Also applies if the participant states that they have a specific skill related to their dream job. Any experience, knowledge, or skills mentioned have to directly relate to their job of interest. The participant provides their job of interest in answer to “What type of job do you hope to get after you graduate?”

- Motivation

The participant expresses being ambitious, persistent, determined, or related terms.

- (SC) Intrinsic

The participant expresses that their passion or interest is an asset that will help them succeed in their dream job.

- (SC) Extrinsic

The participant states that their desire for wealth, prestige, or fame will contribute to their success.

- Intelligence

The participant cites their intelligence as an asset that will help them succeed in their dream job, mentions having a high processing speed, being good at recognizing patterns, being good at learning, critical thinking, or being good at memorizing things.

- Detail orientation

The participant identifies as being detail-oriented or expresses being good at detail recognition.

- Executive functioning
  - (SC) Focus

The participant cites their ability to focus or hyperfocus on a task as a skill that will aid them in their dream job.

- (SC) Reliability

The participant expresses having skills related to being on time, getting work done on time, or staying organized.

- Patience

The participant expresses being calm or patient.

- Social communication
  - (SC) Social skills

The participant expresses being able to work in a team, being charismatic, performing well in interviews, or being good at other social skills.

- (SC) Empathy

The participant cites their compassion or ability to understand the emotions of others as a skill that will help them in their career. This includes related terms such as sympathy and compassion.

- (SC) Written Communication

The participant expresses being good at writing or written communication.

- Work Ethic

The participant cites their work ethic or their ability to work hard as a skill that will help them in their career.

**Question to code: What challenges might you face getting or keeping your dream job?**

- Discrimination

The participant expresses concern about being treated unfairly for being autistic, having another disability, or other factors.

- Motivation

The participant expresses concern with regard to their level of motivation.

- Psychological difficulties

The participant expresses concern in regards to psychological difficulties not directly related to autism (i.e.: not part of the diagnostic criteria in DSM-5). Specifically, when the participant reports feeling anxiety or panic, or feeling depressed.

- Competition

The participant expresses concern about their chances of getting the job, specifically due to competition or lack of jobs in the field

- Executive functioning
  - (SC) Focus

The participant cites having difficulty focusing as a potential challenge.

- (SC) Organization

The participant expresses concern regarding being able to be on time, get work done on time, or stay organized.

- Social communication
  - (SC) Social skills

The participant expresses concern regarding their ability to work in a team, performing well in interviews, or other social skills.

- (SC) Empathy

The participant expresses concern with regard to being able to understand others’ emotions. This includes related terms such as sympathy and compassion.

- (SC) Written Communication

The participant expresses concern with regard to their writing ability or written communication.

- Financial Problems

The participant expresses concern with regard to financial stability: their ability to pay for college, support themselves, or other financial concerns.

- Academic Issues

The participant expresses concern about their ability to obtain a degree or relevant certifications.

- Work Ethic

The participant expresses concern with regard to their work ethic or their ability to work hard enough.

**Question to code: What work-related skills have you developed so far in university?**

- Knowledge relevant to the profession

The participant has gained knowledge relevant to their job of interest, which they provide in answer to “What type of job do you hope to get after you graduate?”

- Executive functioning
  - (SC) Focus

The participant cites having difficulty focusing as a potential challenge.

- (SC) Reliability

The participant has learned to be better at being on time, getting work done on time, or staying organized.

- Social Communication
  - (SC) Social skills

The participant states that they have gotten better at working in a team, performing well in interviews, or other social skills.

- (SC) Empathy

The participant cites their compassion or ability to understand the emotions of others as a skill they have obtained will help them in their career. This includes related terms such as sympathy and compassion.

- (SC) Writing

The participant states that their writing has improved throughout their time in college.

- Nothing

The participant expresses that their time at college was not useful.

**Question to code: What is your major/course of study?**

- STEM
  - SC: Social Sciences & Social Work
    - SSC: Psychology
  - SC: Medical (ex.: nursing, health sciences)
- Arts, Humanities & Linguistics
- Education
- Business
- Liberal Arts or Undecided
- Other

**Question to code: What goals do you hope university will help you achieve?**

1. Academic progression

SC: Graduate degree

SC: field related skills/Advancement of field

SC: Knowledge acquisition- participant indicated gaining knowledge related to academic skills

1. Career prospects

SC: Field specific (stating which career)

SC: Networking (developing field specific connections)

SC: Non field specific (stating general career milestones)

SC: Knowledge acquisition- participant indicated gaining knowledge related to career skills

1. Personal development

SC: Organisation

SC: Independence

SC: Self advocacy

SC: Self-fulfilment

SCC: Recognition/Success

SCC: Happiness

SC: Knowledge acquisition- participant indicated gaining knowledge related to one’s self

1. Interpersonal

SC: Socialisation

SC: Relationships

SC: Communication

1. Community oriented reasons (improving prospects for marginalised groups or wider community)
2. Financial reasons

SC: Paying debt

SC: Acquiring money from work

1. No response
2. Other (Mutually Exclusive)

**Question: What work-related skills have you developed so far in university?**

1. Career skills
   1. Field specific
   2. Non field specific
   3. Knowledge acquisition- participant indicated gaining knowledge related to career skills
2. Academic skills
   1. Field specific
   2. Non field specific
   3. Knowledge acquisition- participants indicated gaining knowledge related to academic skills
3. Personal development
   1. Life skills
      1. Stress management
   2. application of skills to real world
   3. leadership(e.g. Self advocacy)
   4. expression
      1. Creative
   5. Improved executive functioning

i.  Reliability- participant learned about time management or staying organized

ii. Focus - participant learned about multitasking, scheduling, or working hard to achieve goals

1. Interpersonal
   1. Socialisation/Communication
   2. Teamwork
2. N/A (question not applicable)
3. None- no work-related skills were developed
4. Other (mutually exclusive)

**Appendix B**

Table B

*Autistic Students’ Goals in Attending University (n = 84)*

| Code |  |
| --- | --- |
| Career Prospects  Academic Progression | 67%  61% |
| Interpersonal Connections | 35% |
| SC: Socialization | 18% |
| SC: Relationships | 11% |
| Personal Development | 33% |
| SC: Self-fulfillment | 19% |
| Community-oriented (e.g., Help Oppressed) | 15% |
| Financial | 8% |

*Note.* These questions were just asked of autistic participants recruited through snowball sampling.

SC: Indicates sub-code for the major code it is under.

**Illustrative Quotes**

**Question participants were responding to: What goals do you hope university will help you achieve?**

1. Lol. Degrees are just how the bourgeoisie launder privilege. I'm just here to jump thru ableist hoops for a piece of paper and to get personal connections along the way. I already am a good engineer with several inventions & publications under my belt, and it's hard not to feel like I'm wasting my time, especially with impending ecological collapse that I should be out working on right now!
2. I hope that I will be able to get a degree, so that I can be able to work in a medical lab. I also hope that university will help me improve my social skills.
3. The ability to work towards goals in a disciplined manner, better socializing, ability to participate in groups/group dynamics, ability to take notes, better/refined attention, a sense of routine and accomplishment... writing, speaking better, thinking better (more clearly), gaining literacy and knowledge. And maybe something to put on my CV??
4. To be able to learn skills to help me get a successful job into the future while also being able to appreciate life with a great work-life balance. As it is important to appreciate the beauty of life which many people take for granted.
5. I aspire to become a social worker and help underprivileged children/teens. While at college I hope to be able to become more sociable and ultimately become more at ease around people (harder then I had expected)
6. I hope to continue acting as an autistic autism researcher and ultimately transition (presumably with at least one post-doc in between) from my graduate program to an academic faculty position that will allow me to do research.
7. I think my "goals" are different to most of my fellow students at X and in Australian universities in general. I went back to study after bad experience in an unsuitable job. I had autistic burnout. I was severely exhausted. I have already done a Bachelor degree in music, but because of my hearing loss I didn't want to work in that area.
8. I came back to uni for my mental health. I wanted to feel more alive, to connect with myself and my interests and to be able to use my brain in ways that click. I have been interested in linguistics for a long time and have dreamed of studying it. This reason for studying might seem selfish to some but, when I am connected to learning and can get into flow, I can also connect better with those around me and can usually be a better person. My hope is that I will be able to find ways to help people with the skills I learn, even if not through paid work.
9. To have a career that is a mixture of academic and community based research. I would love to have an adjunct professorship in one of the universities in my city and possibly start my own educational consulting company promoting diversity in education.
10. Getting a degree, assuring a level of financial stability in the future, obtaining social skills to further maneuver in life and more specifically the career world.
11. I want the credentials to back me up while I upend a decent chunk of autism research. Also to be a teacher.
12. I hope to be able to do a better job of communicating verbally with adult staff, as they are there to help me when there is a problem and help me to succeed. I struggle with looking people in the eye and have slow processing speed, so I want to do a better job of voicing my concerns independently on a university level.
13. Gain knowledge in my interests and passions to get into a career that incorporates my passions, which are autism advocacy and teaching autistic students. I also plan on opening up a neurodiversity club on my campus, which would be the first of its kind in the State of Minnesota.
14. Establish my research career, allow me to create group co-mentorship networks for autistic and neurodivergent students, be an autistic researcher doing autism research!

**Appendix C**

Table C

*Employment-Related Skills Autistic Students Reported Learning at University (n = 84)*

| Code |  |
| --- | --- |
| Personal Development  SC: Improved Executive Functioning | 57%  29% |
| SC: Life Skills | 27% |
| SC: Leadership skills | 14% |
| Interpersonal Skills | 32% |
| SC: Socialization | 30% |
| Career-Specific Skills | 27% |
| SC: Not Field Specific | 19% |
| SC: Field Specific | 11% |

*Note.* These questions were just asked of autistic participants recruited through snowball sampling.

**Question participants were responding to: What work-related skills have you developed so far in university?**

A) Uhh‚ A tough one. The thing is, my university education strongly correlates with my autism self-discovery journey, and of course figuring out my autism and my reactions and motivations help me function better in the everyday life too. I don't hate myself that much any more, I guess, I'm learning to forgive myself for my shortcomings while trying to improve still.

B) I am not sure. I don't usually like thinking of things as work skills and non-work skills. I usually prefer units I take to not be focused on learning work skills because when they are focused on "work skills" I usually find it a lot less interesting, and thus, difficult. I usually like learning from theory first and learning how I can do things in the real world later. With a good general understanding of how a system (e.g. the languages of the world) works, I usually feel much more grounded and ready to learn what I can do to help others in the real world with my knowledge.

C) I've been able to hone my time management and critical thinking skills, and have become much more confident interacting with people while at college.

D) I don't know if I've learned any specific work-related skills in college. My writing has improved. But I'm not sure what else.

E) On the other hand, my career ambitions are mostly academia-related, so of course being in the university helps, just by knowing the right people, the right jargon, the inner workings of the field. I'm not sure if this answers your question or not, but that's the best I have right now.

F) Interacting in a professional manner and self advocating

G) Knowing to avoid romantic relationships with women you work with.

H) I've taken bullshit classes to learn mathematical methods of analysis I won't use in my career. I learned the bare minimum of drafting/CAD. Nothing about actual engineering methodology.

I) It's too early to answer this question more accurately.

J) Well, I learned to go speak to my professors at the end of class.

K) I guess I'm better at planning, and at learning to be on time to appointments because of getting ready for classes. I've also learned the value of a consistent work ethic and also self-teaching. (I had to learn how to self-teach because I strongly dislike(d) most of my college courses). I have learned that I love learning and that school is not the best avenue of learning for me per se, it's just socially convenient..

L) In undergrad I developed research skills that I use in my current job. I also worked as an RA which increased my interpersonal skills, which I use to facilitate networks of autistic students.

M) My executive function issues have gotten better, I've learned how to talk to authorities to work around my autism and other disabilities so that they are strengths not weaknesses or at the very least alternate methods.

**Appendix D**

**Question participants were responding to: What skills do you have that could help you succeed in your dream job?**

A) Organization, attention to detail, biology knowledge, laboratory techniques

B) skill at conceiving 3d environments/interactions in my head, hyperfocusing on problems, approaching problems from unique perspectives

C) Ability to process information very rapidly and notice patterns. Good teaching, mentoring and writing skills.

D) When I care about something I will set my mind to it, and do it even if it's hard, or bloody painful.... I am creative, intelligent, resourceful, detail oriented, and I am damn good at picking up insights about my field if I want to! I am a great researcher!

E) Music theory, writing, charisma

F) detail-orientated, organised, thorough, dedicated, loyal, charming, supportive colleague, solution-orientated.

G) Ability to keep focus, working with other people, speaking professionally

H) Public speaking, writing, organization, content knowledge, compassion

I) Motivation, I have gone through hard times and I know how these children/teens feel there are many who have no one in there life who cares and that makes me sick to my stomach. I just want to make a difference

J) I'm an adept public speaker, which helps in conferencing but also in teaching. I have an analytical thinking pattern, which helps me recreate my learning journey, and I can recall what was that thing I didn't understand and then how I figured it out, which is an immense help in teaching. Also, I have writing skills, not so much in English late at night, but still

K) Attention to detail, observant, good writing skills, empathetic toward pets and their owners, methodical, always double-checking everything before finalizing.

L) I am good at writing and understanding complex ideas.

M) Excellent verbal communication skills, personal experience struggling with the education/mental health system, counseling skills (e.g. active listening), dedication

N) Research experience, highly organized, special interest, hyperfocus, curiosity, and I want to do autism research and I am autistic, so I have a unique insider perspective.

O) I am amazing at rady maps, very teck-savvy, good at analyzing and comparing data, good at reading all types of graphs, & I am very good at writing.

P) Knowledge/familarity, empathy, leadership, community-building, writing, social media

Q) Experience, as well as training in how to not make decisions for other autistic people based on my experiences alone. I'm also good at observing behavior related to neurodivergent people to see what could happen and how to solve problems before the arise

R) Well, because of infodumping I am fantastic at synthesizing vast amounts of expert level information into (relatively) short, easy to understand bits of information. I love the challenge of being asked questions, it gets my brain hyped up and excited. I don't have to worry about how to interact with people "properly" if theyre asking me questions I know the answer to, or we're dicussing topics I'm knowledgeable on or interested in.

S) I can't do my dream job It's not possible

T) My skills i developed at university

U) I already have the ability to sit down and teach myself various computer programming languages since I was a young teenager

V) creativity

W) Good knowledge of autism, good knowledge of research methods, adequate programming skills, etc.

X) I am an quick thinker and a rather focused workoholic

Y) Strong biology knowledge, exceptional programming skills for a biology major, thorough and meticulous approach to problems, good at writing resumes/cover letters/LinkedIn, adequate interviewing skills

**Appendix E**

**Question participants were responding to: What challenges might you face getting or keeping your dream job?**

A) burnout, lack of concentration, discrimination, anxiety

B) Social anxiety during interviews.

C) ableist institutions, capitalism, depression due to ableist capitalism

D) Impaired executive functioning: with multiple demands in my job from teaching, research and admin, I struggle to prioritise.

E) Probably the social connections - being a freelance artist requires a lot of social media followers and I am not really sure how to gain them, I dislike stooping to tactics like baiting demographics, or making generic art, or pandering to fans etc... it feels lowly and shallow. IDK. I also think that, given time constraints and the amount of energy that school requires I have little time to devote to developing a work ethic for art and writing that is not school related

F) Shortage of academic positions, potential prejudice against autistic people, stressful nature of academic work ("publish or perish"), need to get research grants, etc.

G) Oooh. People. Networking. Figuring out how the publishing system works, who can you approach for help and how to ask them for it, what to say to people in different situations. Carrying on an adult, two-sided conversation with regular turn-taking. Eye contact. Yeah. People stuff.

H) They might not want to give me a chance once they find out I have autism

I) The fact that I basically can't *do* anything as and when I want to because of my catatonia. I am very slow at producing anything and I get overloaded by very ordinary levels of demand.

J) Myself.

K) Social anxiety, communication misunderstandings, sensory issues

L) I struggle to explain my thoughts to other people.

M) Prejudice against Autistic people (people might not trust an Autistic counselor or teacher to be competent at their job), dealing with difficult clients/students, dealing with rules that I disagree with and get in the way of helping people

N) Finishing my degree

O) I struggle slightly with career fairs and interviewing. I'm not as smooth or polished as I wish I was. I also struggle with being on time and getting enough sleep.

P) Ableism, burnout, overload, self-doubt

Q) 1)College (mostly getting a high gpa; like a 3.8 or above), 2)Money (got to have money to do anything). 3)Procrastination/Hiding in my room (I like to spend most of my time in my room, watching YouTube videos & napping).

R) architecture is a competitive field, with no prior job experiance, im likely not going to be one of the top competitors and may end up job less. Also Architecture is based on the Economy and right now the economy is not doing that amazingly. Lastly I think my poor social skills could play a big factor in potentially not being hired or making the connections I need to achieve my goals

S) Lack of job opportunities, money, inability to drive/lack of public transit, job anxiety (almost all paid jobs give me severe anxiety, even without any other issues), not enough work experience maybe, not being sure where to find the job (can't exactly look on indeed.com for this shit), jobs being in areas i can't get to/work in, lack of money to reloate/buy appropriate clothes/etc

T) I can be a slow learner, although when I do learn something, I really know it and will do it right. There are also people who think I can't be an interpreter because I am autistic, due to my sensitivity to sensory input like light and sound. I get told that I can't just leave the room the way I can in school when I'm on the job. But I think that I will be able to find a balance between making my work accessible to me and also knowing when to be professional and deal with it until I can unload later.

U) Getting someone to carve stuff out based on my strengths

V) the job interview or assessment centers, a "bad" CV because I took so long for my studies, prejudices, only being able to work parttime

W) Sometimes I do not know what to say to someone, I struggle with eye contact (too much or not at all), I worry that even if I don't disclose people will know I'm autistic because of my mannerisms.
